# Adherence to the Colombian guideline on congenital toxoplasmosis

**DOI:** 10.7705/biomedica.7590

**Published:** 2025-09-22

**Authors:** Sara Correa-Pérez, Mauricio Daniel Carrascal-Petro, Catalina Arango-Ferreira, Claudia Patricia Beltrán-Arroyave, Javier Mauricio Sierra-Abaunza

**Affiliations:** 1 Grupo en Investigación Clínica en Enfermedades del Niño y del Adolescente - Pediaciencias, Facultad de Medicina, Universidad de Antioquia, Medellín, Colombia Universidad de Antioquia Facultad de Medicina Universidad de Antioquia Medellín Colombia; 2 Departamento de Pediatría, Hospital San Vicente Fundación, Medellín, Colombia Departamento de Pediatría Hospital San Vicente Fundación Medellín Colombia; 3 Departamento de Infectología Pediátrica, Hospital San Vicente Fundación, Medellín, Colombia Departamento de Infectología Pediátrica Hospital San Vicente Fundación Medellín Colombia

**Keywords:** toxoplasmosis, prenatal diagnosis, toxoplasmosis, congenital, public health, advance directive adherence, guideline adherence, practice guideline, toxoplasmosis, diagnóstico prenatal, toxoplasmosis congénita, salud pública, adhesión a las directivas anticipadas, guía de práctica clínica

## Abstract

**Introduction.:**

Congenital toxoplasmosis is associated with high morbidity and mortality in the neonatal period. Despite the existence of a Colombian clinical practice guideline for the diagnosis and treatment of gestational and congenital toxoplasmosis, adherence to its recommendations remains unknown.

**Objective.:**

To evaluate adherence to the Colombian clinical practice guidelines of congenital and gestational toxoplasmosis in two hospitals in Medellin during 2016-2020.

**Materials and methods.:**

We conducted a descriptive study in children under one year of age with suspected or confirmed congenital toxoplasmosis, treated at two hospitals in Medellin between 2016 and 2020. We used proportion measures to assess adherence to the clinical practice guidelines recommendations.

**Results.:**

Two hundred and forty-seven children were included; 17% had a confirmed congenital toxoplasmosis diagnosis. Adherence to the different clinical practice guidelines recommendations was variable. To diagnose gestational toxoplasmosis, immunoglobulins G and M serologies were ordered in 85.4% of the cases. Use of other diagnostic tests - depending on the clinical situation- ranged from 20 to 41.5%; amniocentesis was indicated in 42.9% of pregnant women, whereas IgM, IgG, and IgA were ordered for 50.6% of newborns. Spiramycin was prescribed to 68.8% of mothers diagnosed with gestational toxoplasmosis. Among patients diagnosed with congenital toxoplasmosis, 80.9% presented clinical manifestations; the most common were those of the central nervous system. The highest adherence to the clinical practice guidelines was observed for the treatment of congenital toxoplasmosis (96.7%).

**Conclusions.:**

Adherence to the recommendations of the Colombian clinical practice guidelines for congenital and gestational toxoplasmosis is variable, likely due to implementation barriers, such as limited dissemination, insufficient training or updates for healthcare personnel, and challenges in accessing diagnostic studies and ensuring follow-up. We recommend developing institutional and government policies to provide periodic updates to prenatal care and newborn care personnel.

## Resumen

**Introducción.:**

La toxoplasmosis congénita está asociada con una gran morbimortalidad durante el periodo neonatal. Aunque existe una guía de práctica clínica colombiana para el diagnóstico y el tratamiento de la toxoplasmosis gestacional y congénita, se desconoce el grado de cumplimiento de sus recomendaciones.

**Objetivo.:**

Evaluar la observancia de las recomendaciones de la guía colombiana de práctica clínica para la toxoplasmosis congénita y gestacional en dos hospitales de Medellín entre el 2016 y el 2020.

**Materiales y métodos.:**

Se desarrolló un estudio descriptivo en menores de un año con sospecha o diagnóstico confirmado de toxoplasmosis congénita en dos hospitales de Medellín entre el 2016 y el 2020. Se usaron frecuencias relativas para evaluar el cumplimiento de las recomendaciones.

**Resultados.:**

Se incluyeron 247 niños; el 17 % fueron diagnosticados con toxoplasmosis congénita. La observancia de las diferentes recomendaciones de la guía de práctica clínica colombiana fue variable. Para el diagnóstico de toxoplasmosis gestacional, se evaluaron las concentraciones de inmunoglobulinas G y M en el 85,4 % de las pacientes. Las recomendaciones sobre otras pruebas diagnósticas se acataron del 20 al 41,5 % de los casos, según las diferentes circunstancias clínicas. Se practicó amniocentesis al 42,9 % de las mujeres gestantes con indicación y se ordenaron serologías de IgM, IgG e IgA al 50,6 % de los recién nacidos.

Se prescribió espiramicina al 68,8 % de las madres con toxoplasmosis gestacional. El 80,9 % de los pacientes con toxoplasmosis congénita presentó manifestaciones clínicas; aquellas relacionadas con el sistema nervioso central fueron las más comunes. La mayor observancia de la guía de práctica clínica colombiana (96,7 %), fue respecto al tratamiento de la toxoplasmosis congénita.

**Conclusiones.:**

La observancia de las recomendaciones de la guía de práctica clínica colombiana es variable y depende de las barreras para su implementación, como la falta de difusión de la guía, la falta de actualización del personal de salud y las dificultades para acceder a estudios de diagnóstico y a un seguimiento adecuado. Se recomienda el desarrollo de políticas institucionales y gubernamentales que garanticen la actualización periódica del personal encargado del control prenatal y de la atención al recién nacido.

Toxoplasmosis is a zoonotic disease caused by the parasite *Toxoplasma gondii,* with variable global prevalence (40-50%), depending on climate conditions and risk factors such as exposure to cat feces, raw or non-processed meats, uncooked vegetables, untreated water, or residence in rural areas ^1^^,^^2^. A higher infection burden is present in low-income countries, with a reported overall prevalence of 47.1% in Colombia ^3^.

Gestational toxoplasmosis is generally asymptomatic, but its screening is required due to the severity of congenital infection, which is responsible for almost 65% of the estimated 1.9 million disability-adjusted life years (DALY) ^4^.

In Colombia, more than half of pregnant women (50-60%) have IgG antibodies against toxoplasmosis, indicating high exposure and circulation within the country ^5^. The rate of mother-to-child transmission varies according to the trimester of maternal primary infection and the absence of gestational toxoplasmosis treatment ^6^.

Congenital toxoplasmosis has a global incidence ranging from 0.5 to 3.4 per 1,000 live births ^5^^,^^7^. In Colombia, the estimated incidence is 2-10 per 1,000 live births ^8^^,^^9^. This condition is a multisystemic disease with a high morbidity and mortality burden during the perinatal period, causing prematurity, hearing impairments, chorioretinitis, hydrocephalus, and neurological sequelae, among others ^4^^,^^8^. Ocular toxoplasmosis is the second leading cause worldwide of congenital blindness, with up to 75% of untreated neonates developing chorioretinitis and up to 50% experiencing long-term neurological sequelae ^4^^,^^10^^,^^11^. However, subclinical disease in neonates occurs in 75% of cases, with symptoms that may manifest many years later ^4^.

Several strategies have been found to reduce mother-to-child toxoplasmosis transmission. Primary prevention includes education on risk factors, and serological and follow-up screenings through pregnancy. Secondary prevention involves timely diagnosis and treatment of gestational toxoplasmosis, which can reduce vertical transmission by up to six-fold ^12^. Finally, proper interpretation of neonatal studies is necessary ^2^.

From this evidence-based approach, the Colombian clinical practice guideline was established in 2013. This guideline has good methodological quality and applicability, as demonstrated in a study comparing international guidelines on gestational and congenital toxoplasmosis ^13^. However, information on adherence and impact of these recommendations remains limited, as reported in a recent literature review in the country ^14^.

The objective of this study was to evaluate adherence to the Colombian clinical practice guideline for the diagnosis and treatment of gestational and congenital toxoplasmosis, to describe perinatal care practices, and to identify strengths and weaknesses in the management of the mother-infant dyad at two high-complexity neonatal care institutions in Medellin.

## Materials and methods

We conducted a descriptive cohort study classified as "no risk" according to Resolution 8430 of 1993 from the *Ministerio de Salud* of Colombia. It was approved by the *Comité de Ética* of the *Universidad de Antioquia* and both participating hospitals. Patients were selected from medical records that reported the corresponding International Classification of Diseases, 10^th^ revision (ICD-10) diagnostic codes related to gestational or congenital toxoplasmosis.

We included infants under twelve months of age with suspected or confirmed congenital toxoplasmosis, treated after birth or during outpatient follow-up at two referral hospitals in Medellin between 2016 and 2020. The sample was convenience-based and included those who met the eligibility criteria during the study period. We excluded patients not evaluated by the pediatric infectious disease service. Electronic medical records served as the primary data source. Data collection forms included sociodemographic, clinical, and other variables required to evaluate adherence to the clinical practice guideline ^2^.

Categorical variables were analyzed using relative and absolute frequencies. For quantitative variables, normality was assessed using the Shapiro-Wilk test. For normally distributed variables, we estimated means and standard deviation; otherwise, medians and interquartile ranges (25-75%) were calculated.

Data was analyzed according to the clinical practice guideline recommendations. Maternal serological screening with initial IgG and IgM was used to determine the applicability of the recommendation for each clinical scenario ^2^:

1. Susceptibility to primary infection is defined by negative IgM and IgG serology.
2. Acute infection (seroconversion) is detected by positive IgM in previously seronegative individuals, or positive IgM and negative IgG, followed by IgG seroconversion after two weeks.
3. Recent infection is indicated by IgM and IgG positivity, low IgG avidity test before 16 weeks of gestation, or positive IgA after 16 weeks, and
4. Chronic infection is confirmed by initial negative IgM and positive IgG, or positive IgM and IgG with high IgG avidity test or negative IgA.

Adherence to recommendations was evaluated using the specific indicators proposed in the same clinical practice guideline ^2^. For other recommendations, relative frequencies were estimated for each clinical scenario.

## Results

**Figure 1:**
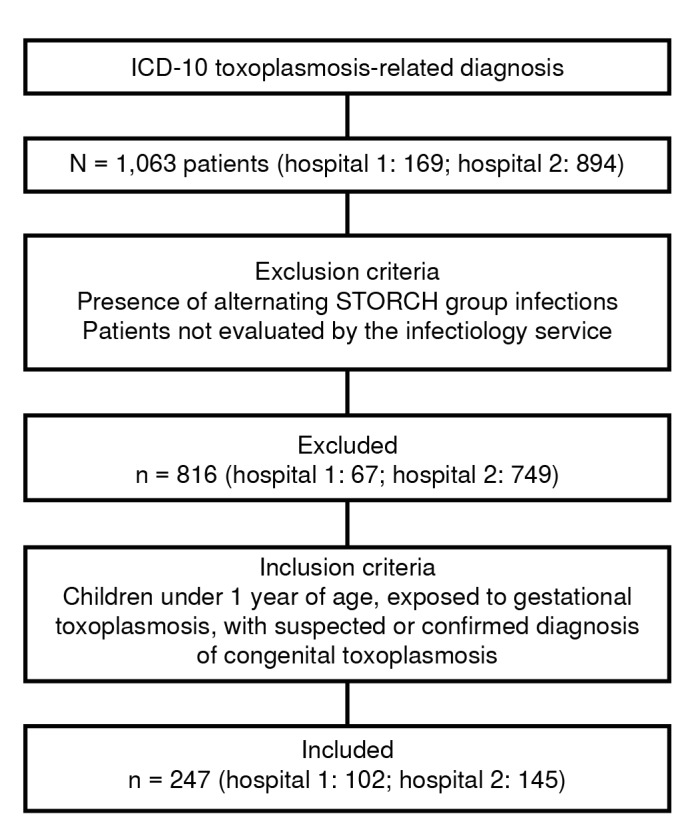
Recruitment flowchart of patients with suspected or confirmed congenital toxoplasmosis in two tertiary-level hospitals in Medellin, Colombia

A total of 247 patients were included (figure 1). Sociodemographic, clinical, and serological data of the included patients are shown in table 1. Adherence to the clinical practice guideline recommendations varied depending on the clinical scenario, as described in table 2. No primary prevention measures for gestational toxoplasmosis were found in the medical records.

ICD-10: International Statistical Classification of Diseases

**Table 1:** Sociodemographic, clinical, and serological characteristics (N = 247)

Variable		n	%
Pregnant individuals			
	With prenatal care	246	99.6
	Prenatal care entry (trimester)		
	1^st^	144	58.5
	2nd	57	22.3
	3^rd^	16	6.3
Number of prenatal visits		7	(5-8)*
*Toxoplasma gondii* transmission risk factors**		74	30
Initial maternal serological screening			
	IgG (-), IgM (-)	41	16.6
	IgG (+), IgM (+)	152	61.5
	IgG (+), IgM (-)	10	4.0
	IgG (-), IgM (+)	8	3.2
	IgG or IgM with missing or indeterminate data	36	14.6
Newborns			
Female		114	46.2
Gestational age			
Preterm		43	17.4
Term		181	73.3
Missing data		23	9.3
Low birth weight (n = 222)		55	24.8
Social security		239	96.8

* Median (IQR)

** Exposure to cat feces, raw or unprocessed meats, or unwashed vegetables; residence in rural areas; lack of access to safe drinking water; gardening activities

**Table 2:** Adherence to the clinical practice guideline recommendations

Recommendation		Performed	Indicated	Adherence
		(n)	(n)	(%)
Prevention and diagnosis of gestational toxoplasmosis				
	Recommendations on maternal risk factors	0	247	0
	Baseline IgM and IgG serology during the first prenatal control	211	247	85.4
	Monthly IgM follow-up (seronegative mothers)	17	41	41.5
	Positive IgG and IgM*	41	144	28.5
	IgG avidity test (before the 16th week)**	30	89	33.7
	IgA (after the 16^th^ week)***	11	55	20
	Negative IgG and positive IgM: two weeks IgG follow-up	2	8	25
Fetal toxoplasmosis				
	Amniocentesis and PCR in the second trimester	30	70	42.9
	Positive IgG and IgM in the first trimester; low-IgG avidity	15	19	78.9
	Positive IgG and IgM in the second or third trimester; positive IgA	0	6	0
	Seroconversion	15	45	33.3
	Fetal ultrasound	222	247	89.9
Gestational toxoplasmosis				
	Spiramycin	55	80	68.8
	Positive IgG and IgM in the first trimester; low IgG avidity	17	19	89.5
	Positive IgG and IgM in the second or third trimester; positive IgA	5	6	83.3
	IgM seroconversion	33	55	60
Congenital toxoplasmosis treatment				
	Pyrimethamine + sulfadiazine + folinic acid	12	16	75
	Positive amniotic fluid PCR	10	10	100
	Central nervous system ultrasound abnormalities	6	10	60
Congenital toxoplasmosis diagnosis				
	IgG, IgM, and IgA testing in newborns with suspected infection	125	247	50.6
	Western blot in newborn with positive IgG and negative IgA and IgM	0	85	0.0
	IgG follow-up in newborn with negative results in all three tests (IgM, IgA, and IgG detected by Western blot)	12	85	14.1
		Congenital toxoplasmosis				
	Pyrimethamine + sulfadiazine + folinic acid or alternative regimen****	41	42	97.6

* Of 152 patients, 8 had positive IgG and IgM, and unknown time of admission.

** Patients with IgM and IgG positive before the 16^th^ gestational week, with indication of avidity test

*** Patients with IgM and IgG positive after week the 16^th^ gestational week, with indication of IgA

**** Alternative regime: clindamycin, sulfadoxine, or azithromycin in combination with pyrimethamine + folinic acid

IgG: Immunoglobulin G; IgM: Immunoglobulin M; IgA: Immunoglobulin A; PCR: Polymerase chain reaction

In compliance with the guidelines, 30.2% (13/43) of IgA tests were performed after week 16, of which six were positive for gestational toxoplasmosis. Additionally, 69.7% (30/43) underwent IgG avidity testing. Upon admission, two of the eight pregnant women with negative IgG and positive IgM had an adequate follow-up. Both seroconverted IgG at two weeks, confirming recent exposure. Subsequently, their two neonates underwent confirmatory testing, including positive cerebrospinal fluid PCR and IgM, and presented central nervous system manifestations.

Amniocentesis was performed on 56 patients. *Toxoplasma gondii* DNA was detected by PCR in 10 (17.8%) samples, while 26 (46.4%) had no clear indication according to the clinical practice guideline. Unfortunately, no details on the PCR test were available.

**Figure 2:**
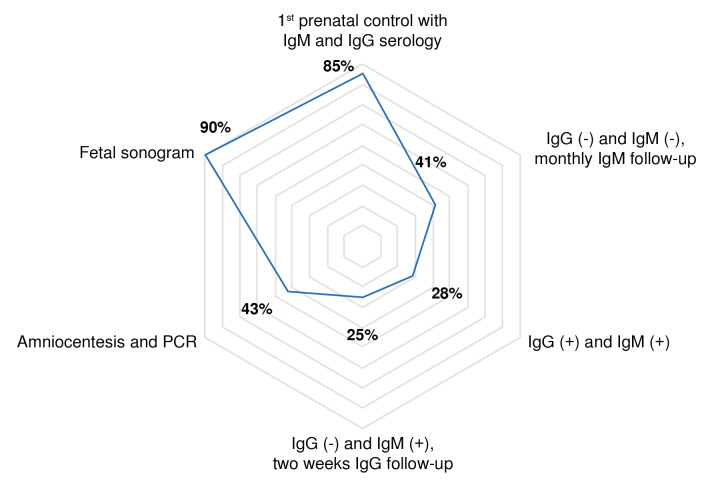
Adherence to diagnostic recommendations for gestational toxoplasmosis

Of the 222 gestational ultrasounds, 43 (19.3%) had abnormal findings. The most frequent were intrauterine growth restriction (n = 29; 13%), ventriculomegaly (n = 5; 2.2%), and brain calcifications (n = 4; 1.8%). Figure 2 displays the adherence percentages to the clinical guideline recommendations for the serological parameters for the diagnosis of gestational toxoplasmosis.

PCR: Polymerase chain reaction

Spiramycin was prescribed to 162 patients, of whom 80 (52.4%) met the clinical criteria for its use. Among those treated, 108 patients received an adequate dose, and 62 (38.2%) initiated treatment opportunely, within four weeks of diagnosis. Causes of inadequate spiramycin treatment included inconclusive diagnosis (n = 12), administrative reasons (n = 7), incomplete treatment duration (n = 7), and patient refusal (n = 1). Seventy-nine patients received spiramycin without a clear indication.

Fifteen pregnant women received fetal toxoplasmosis treatment; 10 had a positive amniotic fluid PCR, two had fetal ultrasound abnormalities related to the central nervous system (calcifications), and three had unidentifiable indications. Of the 162 children whose mothers received spiramycin, 21 (12.9%) had suggestive symptoms of congenital toxoplasmosis either in the central nervous system or the retina. In contrast, 24 (28.2%) out of 85 patients whose mothers did not receive treatment developed clinical manifestations.

Postnatal diagnosis of congenital toxoplasmosis was confirmed in 42 patients (17%). Seroconversion occurred in 3 patients during the first trimester, 11 during the second, and 11 during the third one; in 12 cases, the trimester of infection could not be identified. Five neonates were diagnosed based on their clinical manifestations, all born to mothers with poor prenatal serological monitoring or follow-up.

The diagnostic methods included: positive IgM in 22 (52.4%) patients, positive IgA in 2 (4.8%) patients, positive cerebrospinal fluid PCR in 8 (19%) patients, elevated IgG associated with clinical symptoms and a history of gestational toxoplasmosis in 2 (4.8%) patients, and two or more confirmatory tests for congenital toxoplasmosis in 8 (19%) patients. Physical examination and other diagnostic aids revealed abnormalities in 34 (80.9%) infants, as described in table 3.

**Table 3:** Clinical and laboratory manifestations of congenital toxoplasmosis (N = 42)

Manifestations		n	%
Neurological		28	66.7
	Calcifications	12	28.6
	Hypotonia, seizures	10	23.8
	Hydrocephalus	3	7.1
	Microcephaly	2	4.8
Ophthalmological		25	59.5
	Chorioretinitis	11	26.2
	Retinal hemorrhages	2	4.8
	Inactive lesions	5	11.9
Hematological		5	11.9
	Thrombocytopenia	4	9.5
	Anemia	1	2.4
	Leukocytosis	0	0.0
Hepatic		12	28.6
	Impaired liver function	9	21.4
	Hepatomegaly	2	4.8
Asymptomatic		8	19.1

Among the 34 children with central nervous system or retinal manifestations, 12 (35.2%) were born to mothers who had received spiramycin treatment. Of those with confirmed congenital toxoplasmosis, 11 (26.1%) received pyrimethamine/sulfadoxine/folinic acid during pregnancy.

Six (24.3%) patients were exposed to *T. gondii* during pregnancy and developed clinical symptoms suggestive of infection; however, no paraclinical confirmation was performed. All of them received treatment.

Cerebrospinal fluid samples were ordered for 73 newborns (29.6%) and collected on 69 (27.9%). Analysis showed abnormal results in 23 cases (33%). Out of the 51 (69.8%) PCR samples, 8 (15.6%) were positive for *T. gondii.* Brain imaging was performed on 231 (93.5%) neonates. Auditory evoked potentials were requested for 150 neonates (60.1%), and 73 (29%) underwent the test, with no abnormal findings reported.

Of 42 patients diagnosed with congenital toxoplasmosis, only one did not receive treatment due to early death. Among the 41 treated patients, 21 (51.2%) received first-line treatment with pyrimethamine/sulfadiazine/folinic acid, 4 (9.5%) were treated with alternative regimens such as pyrimethamine/ sulfadoxine/folinic acid or trimethoprim-sulfamethoxazole, and 16 (39%) initially received alternative management while waiting for first-line treatment.

It is important to consider that 12 (28.5%) patients experienced difficulties with authorization, timely delivery, or consistent supply of medications, which led to the use of alternative regimens. Among patients diagnosed with congenital toxoplasmosis, 8 (19%) received corticosteroids; of them, 2 (4.75%) had chorioretinitis, while the remaining cases had unclear indications for steroid treatment.

## Discussion

Evidence has shown that implementation of the clinical practice guideline improves the timely diagnosis and treatment opportunity of gestational and congenital toxoplasmosis, with a reduction in the number of severe cases and sequelae in children ^2^. This study is one of the few that evaluates the implementation of the Colombian clinical practice guideline for toxoplasmosis and found variable adherence -higher for treatment and lower for clinical or serological follow-up- in patients with suspected gestational toxoplasmosis. These findings are likely related to implementation barriers such as limited dissemination, insufficient training or updates for health personnel, and restricted access to diagnostic tests and follow-up care. These results align with those described by Wang *et al.* in their systematic review, which report a 60-70% non-compliance with the general clinical practice guidelines ^15^.

Adherence to the diagnostic recommendations for gestational toxoplasmosis ranges from 20 to 85.4%, with greater difficulty observed in the serological follow-up after the initial IgG and IgM screening. During diagnosis and serological follow-up, we found that a large number of the ordered IgG avidity tests and IgA serologies did not comply with the clinical practice guideline, which can alter the interpretation and lead to diagnostic errors ^16^. Almost 50% of amniocentesis had no clear indications. These practices can cause negative outcomes for the patient and represent unnecessary use of healthcare system resources, as reported by Liu *et al*^16^.

Spiramycin has been shown to reduce the risk of vertical transmission by up to 52% ^7^. During our study, 68.8% of mothers with gestational toxoplasmosis received this treatment. In comparison, Mejía-Oquendo *et al.* reported that only 52.5% of mothers were treated against *T. gondii* infection before the implementation of the clinical practice guideline. However, this percentage changed after its implementation, since all mothers received adequate treatment ^17^. In this study, conducted in Quindío, Colombia, central nervous system or retinal manifestations were observed in 8.7% of patients whose mothers received spiramycin as treatment compared to 28.2% of those whose mothers did not receive treatment ^17^. More than half of the pregnant women who received spiramycin did not meet the criteria outlined in the clinical practice guideline, suggesting a lack of clarity regarding the appropriate indication and timing for initiating secondary prevention ^2^^,^^6^. The Colombian and French clinical practice guidelines recommend that treatment for acute gestational toxoplasmosis should be started early, ideally within the first three to four weeks after seroconversion ^2^^,^^9^^,^^18^^,^^19^. This study identified different treatments initiated later than recommended.

According to the clinical practice guideline, diagnosis of congenital toxoplasmosis is defined by a history of gestational toxoplasmosis and positive neonatal serology ^2^. Adherence to diagnostic recommendations in newborns ranged from 0 to 50%. We highlight that seronegative results after birth do not rule out congenital toxoplasmosis ^20^. Therefore, some studies have established additional criteria to support the diagnosis, including: detailed physical evaluation, positive serology ^6^^,^^21^, IgG serology follow-up ^6^^,^^21^, molecular testing for *T. gondii* identification ^6^^,^^21^, and imaging studies to assess systemic compromise. A postnatal follow-up remains necessary in the first year of life to fully exclude the infection ^4^. Additionally, recent literature does not report Western blot for diagnosis, probably explained by the lack of commercial tests ^6^^,^^21^.

In contrast with the literature reporting up to 85% of patients with congenital toxoplasmosis as asymptomatic ^11^, the present study only found 19.1%. Central nervous system and retina involvement were the most frequent clinical manifestations. These findings can be explained by the fact that one of the two included hospitals is a referral center -where more severe cases are concentrated- and because of the low proportion of mothers (35.2%) who received spiramycin treatment.

The highest adherence (96.7%) was observed in the treatment of congenital toxoplasmosis using first-line schemes or therapeutic alternatives ^6^^,^^12^^,^^21^. This percentage reflects compliance with the clinical practice guideline at high complexity centers, guided by infectious disease specialists. In cases where first-line treatment or alternative regimens were unavailable, trimetoprim/sulfametoxazol was used. This scheme has been described by authors such as Hernández *et al.,* particularly for cerebral toxoplasmosis in developing countries (in the absence of other regimens) due to several advantages, like tolerability, multiple formulations, and easy access ^19^. Additionally, the literature recommends corticosteroid treatment for patients with severe chorioretinitis or high protein levels (≥ 1 g/dl) ^6^ in the cerebrospinal fluid. This scenario occurred in a group of patients in this study, but the clinical practice guideline does not include these indications ^2^.

The literature reports that the time of maternal primary infection determines the risk of transmission and the severity of the clinical presentation in the neonate. In our study, seroconversion was more frequent during the second and third trimesters (more than 50%). However, cross-sectional analyses were not performed to associate seroconversion with the severity of the clinical presentation ^6^^-^^7^.

We identified several weaknesses related to the study's design, including possible data loss, as it relies on information recorded in electronic medical records. Additionally, a risk of selection bias exists as it includes patients assessed by infectious disease specialists in high-complexity centers.

Despite high morbidity and mortality associated with toxoplasmosis in newborns, knowledge gaps persist among pregnant women and healthcare providers regarding prevention, diagnosis, and treatment ^22^. Adherence to clinical practice guideline recommendations for toxoplasmosis was variable. Opportunities to enhance adherence have been identified, especially for the diagnosis and follow-up of pregnant women. Some clinical scenarios circumstances are not included in the clinical practice guideline but could be addressed in future updates. For example, recommendations for the clinical and paraclinical follow-up are not clear for children with suspected or diagnosed congenital toxoplasmosis. Institutions and governments should develop policies to provide periodic updates to prenatal and newborn care personnel ^22^.
